# Evaluation of T2W FLAIR MR image quality using artificial intelligence image reconstruction techniques in the pediatric brain

**DOI:** 10.1007/s00247-024-05968-8

**Published:** 2024-06-18

**Authors:** Usha D. Nagaraj, Jonathan R. Dillman, Jean A. Tkach, Joshua S. Greer, James L. Leach

**Affiliations:** 1 Department of Radiology and Medical Imaging, Cincinnati Children’s Hospital Medical Center, 3333 Burnet Avenue, Cincinnati, OH 45229-3026 USA; 2 Department of Radiology, University of Cincinnati, Cincinnati, OH USA; 3 Philips Healthcare, Cincinnati, OH USA

**Keywords:** Artificial intelligence, Brain, Child, Fluid-attenuated inversion recovery, Gray matter, Magnetic resonance imaging, White matter

## Abstract

**Background:**

Artificial intelligence (AI) reconstruction techniques have the potential to improve image quality and decrease imaging time. However, these techniques must be assessed for safe and effective use in clinical practice.

**Objective:**

To assess image quality and diagnostic confidence of AI reconstruction in the pediatric brain on fluid-attenuated inversion recovery (FLAIR) imaging.

**Materials and methods:**

This prospective, institutional review board (IRB)-approved study enrolled 50 pediatric patients (median age=12 years, Q1=10 years, Q3=14 years) undergoing clinical brain MRI. T2-weighted (T2W) FLAIR images were reconstructed by both standard clinical and AI reconstruction algorithms (strong denoising). Images were independently rated by two neuroradiologists on a dedicated research picture archiving and communication system (PACS) to indicate whether AI increased, decreased, or had no effect on image quality compared to standard reconstruction. Quantitative analysis of signal intensities was also performed to calculate apparent signal to noise (aSNR) and apparent contrast to noise (aCNR) ratios.

**Results:**

AI reconstruction was better than standard in 99% (reader 1, 49/50; reader 2, 50/50) for overall image quality, 99% (reader 1, 49/50; reader 2, 50/50) for subjective SNR, and 98% (reader 1, 49/50; reader 2, 49/50) for diagnostic preference. Quantitative analysis revealed significantly higher gray matter aSNR (30.6±6.5), white matter aSNR (21.4±5.6), and gray-white matter aCNR (7.1±1.6) in AI-reconstructed images compared to standard reconstruction (18±2.7, 14.2±2.8, 4.4±0.8, *p*<0.001) respectively.

**Conclusion:**

We conclude that AI reconstruction improved T2W FLAIR image quality in most patients when compared with standard reconstruction in pediatric patients.

**Supplementary Information:**

The online version contains supplementary material available at 10.1007/s00247-024-05968-8.

## Introduction

Artificial intelligence (AI) methodologies allow for improved magnetic resonance (MR) image quality by applying deep learning-based reconstruction schemes to fully or under-sampled k-space data resulting in improved or preserved image quality in equivalent or reduced acquisition time [1]. However, their use in clinical practice, in particular for magnetic resonance imaging (MRI) of the pediatric brain, has yet to be adequately explored. Such evaluation is needed as these algorithms are trained mostly, or even entirely, on adult datasets [2]. The purpose of this study is to assess image quality and diagnostic confidence of a United States Food and Drug Administration-approved AI MR image reconstruction algorithm in the pediatric brain on fluid-attenuated inversion recovery (FLAIR) imaging for potential clinical use in the pediatric population.

## Materials and methods

This prospective, institutional review board (IRB)-approved, single-site study included 50 pediatric patients undergoing routine, non-contrast clinical brain MRI examinations enrolled between July 2022 and January 2023. Exclusion criteria included age less than 2 years, need for imaging under general anesthesia or sedation, and history of neurologic surgery and/or the presence of intracranial implants. Informed consent was obtained from all participants, and informed assent was obtained, as appropriate.

**Fig. 1:**
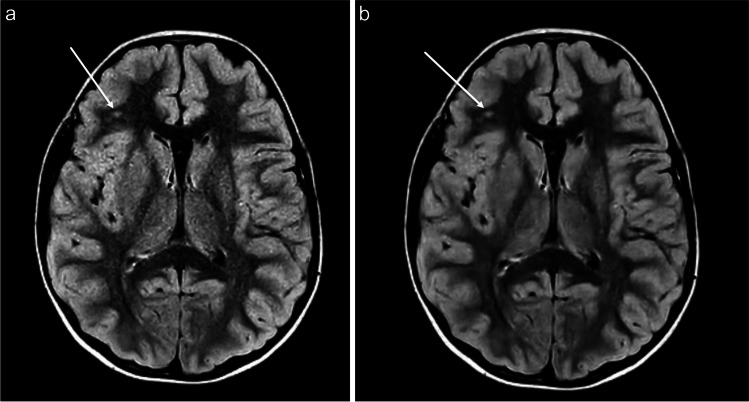
**a-b** Axial T2W FLAIR CS=1.6 brain MR images in an 8-year-old female presenting with repeated episodes of vomiting in the middle of the night without (**a**) and with (**b**) AI image reconstruction (SmartSpeed; Philips Healthcare). The AI image shows a reduction in image noise compared to the standard image reconstruction. There is also a focus of T2W FLAIR hyperintense signal in the right frontal white matter (arrows), doubtful clinical significance however is clearly reproduced on the AI reformat. Note is also made of a cavum septum pellucidum, normal anatomic variant, also accurately reproduced

**Fig. 2:**
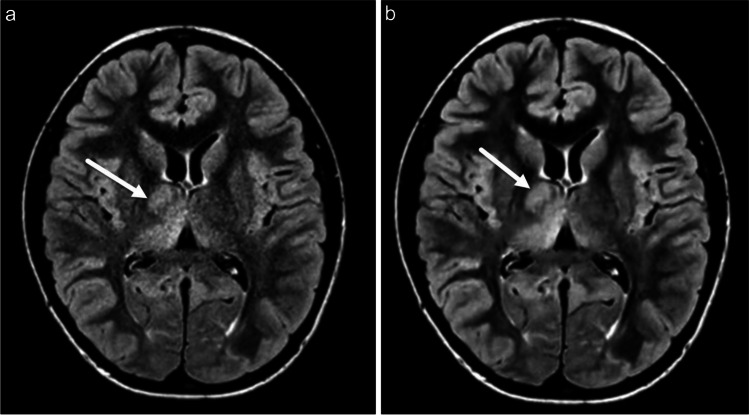
**a-b** Axial FLAIR CS=1.6 brain MR in a 14-year-old female presenting with new onset of headaches and dizziness without (**a**) and with (**b**) AI image reconstruction demonstrates global reduction in image noise and ill-defined signal abnormality in the right thalamus (arrows) suspicious for infiltrative neoplasm, reproduced on AI reconstruction

**Fig. 3:**
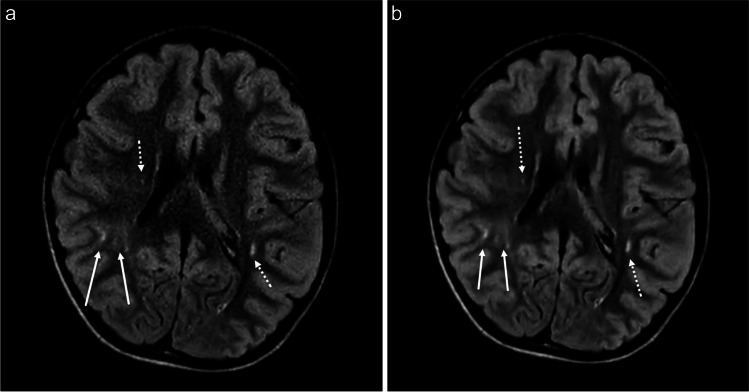
**a-b** Axial T2W FLAIR CS=1.6 brain MR in a 8-year-old male with known diagnosis of tuberous sclerosis with standard (**a**) and AI (**b**) reconstruction demonstrating a dysplastic lesion in the right parietotemporal junction involving the subcortical and transmantle white matter (solid white arrows). There are additional smaller T2W FLAIR hyperintense foci in the bilateral periventricular white matter (dashed arrows), also accurately reproduced on AI reconstruction

All imaging was performed on a single 1.5-Telsa MR scanner (Ingenia; Philips Healthcare; Best, the Netherlands) using the body coil transmit and a 15-channel receive only head coil. Clinical 2D T2-weighted (T2W) FLAIR (repetition time (TR)=11,000 ms, inversion time (TI)=2,800 ms, echo time (TE)=140 ms, acquired in plane resolution=0.79 mm×0.78 mm, 4-mm contiguous interleaved slices; compressed SENSE (CS) acceleration factor=1.6) images from our clinical imaging protocol were reconstructed with both standard CS (medium denoising) image reconstruction and AI “strong” denoising (selected from a 4-point scale provided by vendor: weak, medium, strong, and maximum) algorithm (SmartSpeed; Philips Healthcare, Best, The Netherlands). All T2W FLAIR images (Figs. 1, 2, and 3) were reviewed independently in a dedicated research picture archiving and communication system (PACS) by two study neuroradiologists, both with added qualifications in neuroradiology and one with additional added qualifications in pediatric radiology. The research PACS system used (Merge Healthcare, Version 8.1.6.7) allowed the radiologists to be blinded to patient information and clinical data during the review process; however, the radiologist was not blinded to image reconstruction method during side-by-side review. AI images were evaluated on a 3-point scale (poor, sufficient, excellent) for overall image quality, subjective assessment of signal to noise ratio (SNR), various artifacts, and diagnostic preference. Each sequence was also rated on a 3-point scale to indicate whether AI increased, decreased, or had no effect on image quality compared to the standard reconstruction. Additional imaging features were assessed and compared between the AI and standard reconstruction images and included CSF artifacts, motion artifacts, susceptibility artifacts, gray-white matter differentiation, image sharpness, flow void visualization, and extracranial structure evaluation (Supplementary Material 1). The presence of pathology, normal variants, or lack thereof were also commented on.

**Fig. 4:**
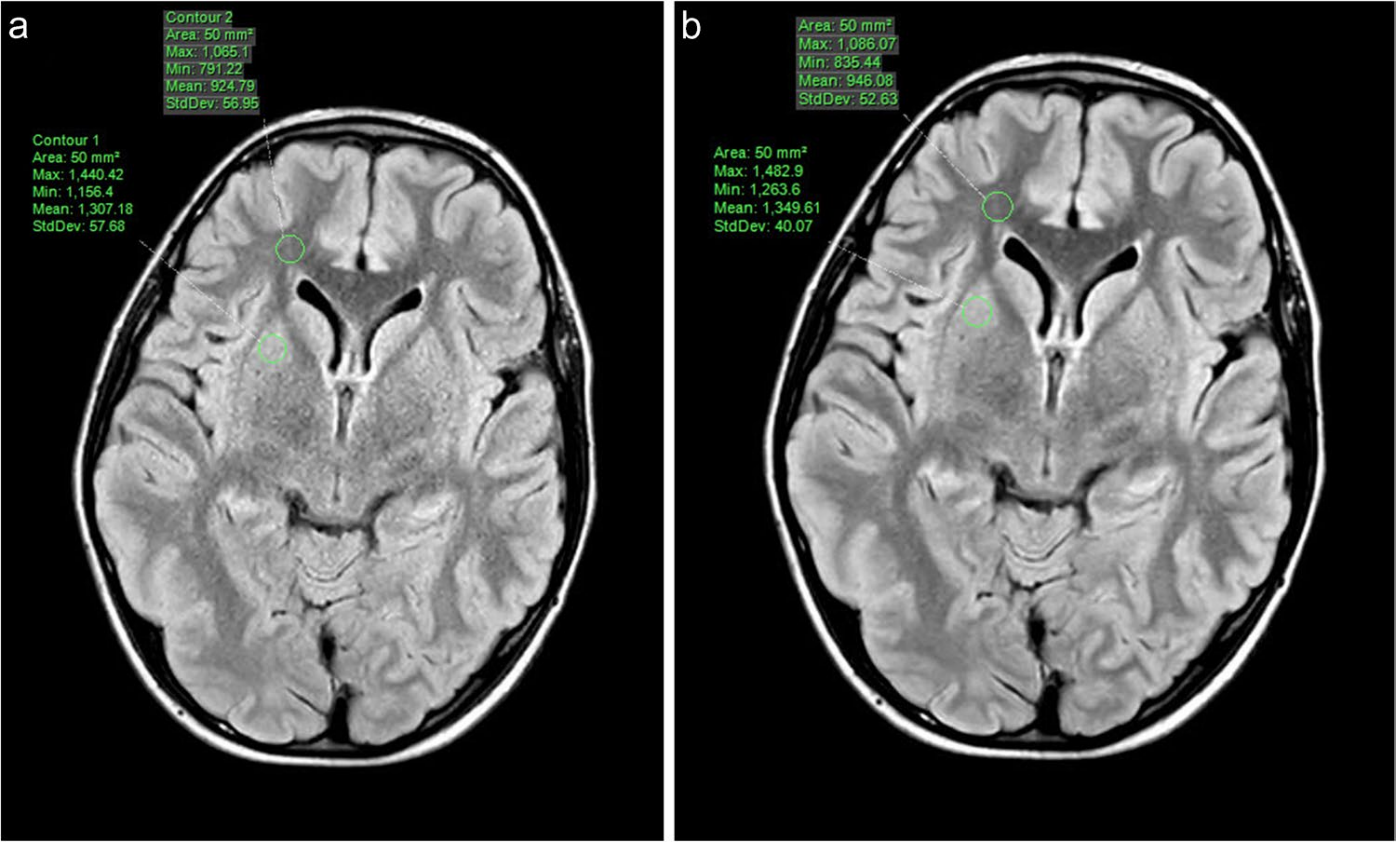
**a-b** Axial T2W FLAIR brain MR images in an 11-year-old male demonstrating circular ROIs placed in the right frontal white matter and right putamen for signal analysis in the standard reconstruction (**a**) and AI reconstruction (**b**) images

Quantitative analysis was performed using Philips IntelliSpace Portal (Version 10.1, Philips Healthcare). Regions of interest (ROIs) were manually placed by one neuroradiologist in the right putamen and right frontal white matter, and the mean and standard deviation of the signal within each ROI were used to calculate apparent signal to noise ratio (aSNR) gray matter, aSNR white matter, and apparent gray-white matter contrast to noise ratio (aCNR) (Fig. 4). aSNR was calculated using the following equation: aSNR tissue=µ tissue/σ tissue. aCNR was calculated using the following equation: aCNR white matter–gray matter=|(µ white matter−µ gray matter)/√𝜎 white matter ^2^+𝜎 gray matter^2^| (µ, signal intensity; σ, standard deviation) [3].

Statistical analysis was performed using Excel (2021, Microsoft) spreadsheet mathematic functions including mean values, standard deviations, value count, and *t* test results. *P*-values less than 0.05 were considered statistically significant. Descriptive statistical analyses of the independent reviews were performed. We did not use kappa coefficients due to unbalanced observation across scores.

## Results

**Fig. 5:**
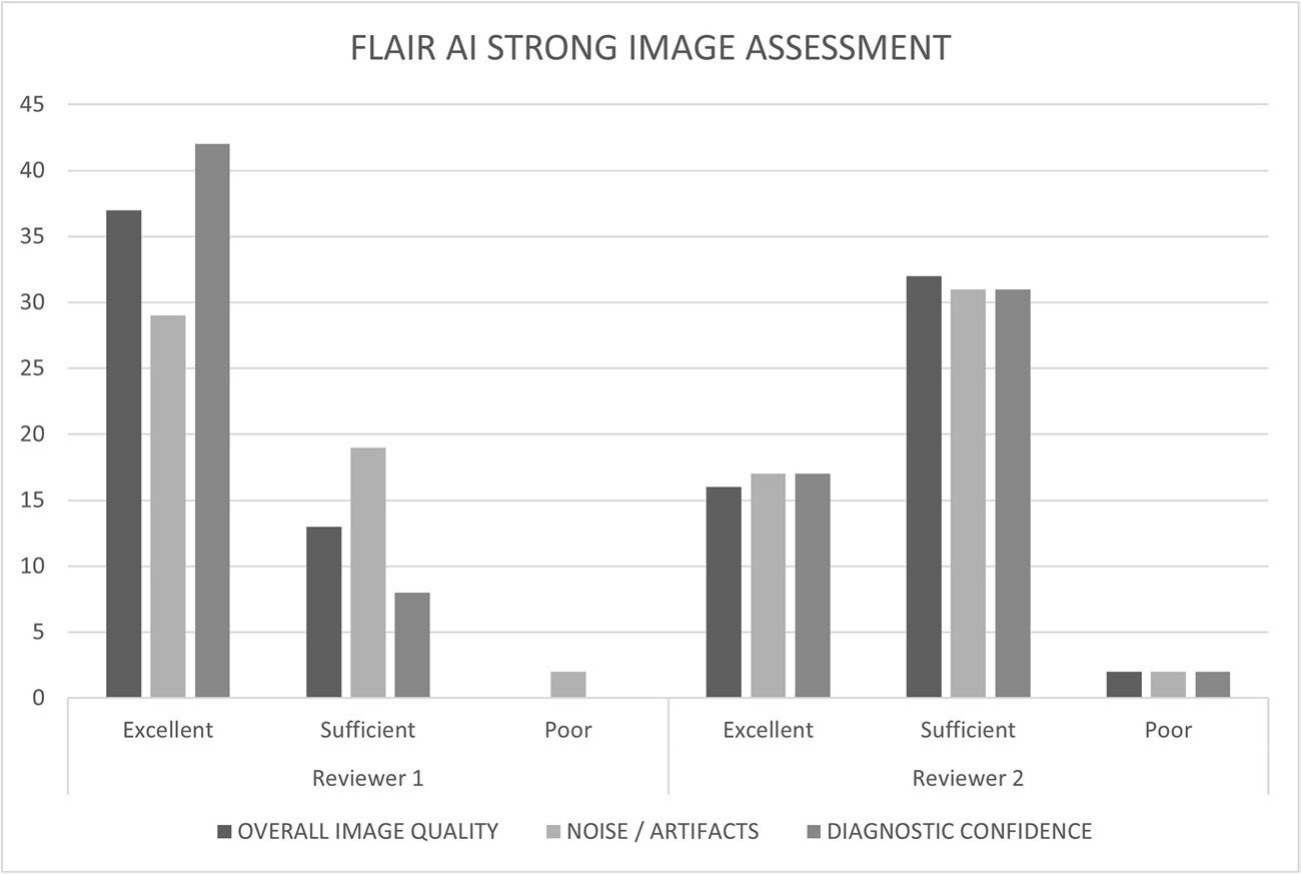
Data from the neuroradiologists assessment of T2W FLAIR AI strong image quality

Images from all 50 patients were reviewed by the two study neuroradiologists to evaluate for concordance. Patient demographics are summarized in Table 1. T2W FLAIR with AI had excellent or sufficient imaging quality in 98% (reader 1, 50/50; reader 2, 48/50) of cases and excellent or sufficient diagnostic confidence in 98% (reader 1, 50/50; reader 2, 48/50) of cases. These and other findings are summarized in Fig. 5. When compared with non-AI, 99% (reader 1, 49/50; reader 2, 50/50) of evaluations submitted reported better overall image quality and subjectively improved SNR with AI. 99% (reader 1, 49/50; reader 2, 49/50) of evaluations submitted reported better diagnostic preference with AI compared with non-AI. When comparing standard versus AI-reconstruction, the degree of artifacts related to cerebrospinal fluid (CSF) pulsation and magnetic susceptibility were the same in 100% (readers 1 and 2, 50/50) of patients. Motion artifacts were the same in 98% (reader 1, 49/50; reader 2, 49/50) of patients. These and other findings are summarized in Table 1.

**Table 1:** Table 1 Patient demographics

Patient age in years (median, IQR)	12 (10, 14)
Sex	42% (21/50) male, 48% (29/50) female
Reasons for exam	Headaches/migraines, 66% (33/50) Neurologic deficits, 10% (5/50) Psychiatric disorder, 10% (5/50) Vomiting, 6% (3/50) Other, 8% (4/50)
T2W FLAIR sequence scan time in seconds (mean±SD)	263.8±1.3

*IQR*, interquartile range; *T2W*, T2-weighted; *FLAIR*, fluid-attenuated inversion recovery

**Table 2:** Table 2 Summarized findings

T2W FLAIR AI vs. non-AI	Reviewer 1 (*n*=50)	Reviewer 2 (*n*=50)	% concordance
Overall image quality	100% (50/50) better	98% (49/50) better 2% (1/50) same	98% (49/50)
Subjective SNR	98% (49/50) better 2% (1/50) same	100% (50/50) better	98% (49/50)
Diagnostic preference	98% (49/50) better 2% (1/50) same	98% (49/50) better 2% (1/50) worse	96% (48/50)
CSF artifacts	100% (50/50) same	100% (50/50) same	100% (50/50)
Motion artifacts	98% (49/50) same 2% (1/50) worse	98% (49/50) same 2% (1/50) better	96% (48/50)
Susceptibility artifacts	100% (50/50) same	100% (50/50) same	100% (50/50)
GM/WM differentiation	36% (18/50) better 64% (32/50) same	88% (44/50) better 12% (6/50) same	36% (18/50)
Image sharpness	54% (27/50) better 46% (23/50) same	100% (50/50) better	54% (27/50)
Flow void visualization	100% (50/50) same	2% (1/50) better 98% (49/50) same	98% (49/50)
Extracranial structure evaluation	66% (33/50) better 34% (17/50) same	10% (5/50) better 90% (45/50) same	44% (22/50)

*T2W*, T2-weighted; *SNR*, signal to noise ratio; *CSF*, cerebrospinal fluid; *GM/WM*, gray matter white matter

Quantitative analyses of signal intensities yielded significantly higher aSNR in both white matter (21.4±5.6) and gray matter (30.6±6.5) with AI reconstruction compared to standard reconstruction (14.2±2.8, 18±2.7, *p*<0.001) respectively. The gray-white matter aCNR was also significantly higher in the AI reconstruction (7.1±1.6) compared to standard (4.4±0.8, *p*<0.001). Values are listed in Table 1.

**Table 3:** Table 3 Standard and AI reconstruction values

	Standard reconstruction	AI reconstruction	*p*-values
Gray matter aSNR	18±2.7	30.6±6.5	<0.001
White matter aSNR	14.2±2.8	21.4±5.6	<0.001
Gray-white matter aCNR	4.4±0.8	7.1±1.6	<0.001

*aSNR*, apparent signal to noise ratio; *aCNR*, apparent contrast to noise ratio

Pathology or normal variants were observed in the standard reconstruction images by the principal investigator in 42% (21/50) of patients and were reproduced in all cases on the AI images; the remaining 58% (29/50) of exams were interpreted as normal and study interpretations were subsequently compared to the official clinical reports to exclude the possibility of overlooked clinically relevant pathology. Pathologies identified included foci of presumed gliosis (*n*=6), Chiari I (*n*=2), mild ventriculomegaly (*n*=1), arachnoid cysts (*n*=1), dolichocephaly (*n*=1), cystic encephalomalacia (*n*=1), tuberous sclerosis (*n*=1), and a right thalamic mass (*n*=1). Normal variants included mega cisterna magna (*n*=2), cerebellar developmental venous anomaly (*n*=1), enlarged perivascular spaces (*n*=1), and cerebellar ectopia not meeting criteria for Chiari I (*n*=1). No additional anomalies were identified on the AI images by either radiologist.

## Discussion

In this study, we observed that AI MR image reconstruction improved overall image quality in clinical brain T2W FLAIR images in most patients. This was accomplished without an increase in scan time and without perceived loss of diagnostic quality or information in this study sample and no additional anomalies were identified. Both qualitative and quantitative assessments in SNR were improved on the AI-reconstructed images compared to standard reconstruction. While no loss of gray-white matter differentiation or image sharpness was appreciated by either reviewer on the AI-reconstructed images for any of the studies examined, there was no strong consensus as to whether or not AI improved these image parameters in this cohort (36% (18/50) and 54% (27/50) respectively).

Use of AI reconstruction algorithms for highly under-sampled k-space data has been described as a potential method to decrease image acquisition time without compromising image quality in proton density of the knee, 2D fast spin echo (FSE) of the hip and shoulder, and 3D T2 turbo-spin echo (TSE) of the lumbar spine MRI [3–6]. Improved image quality with AI reconstruction algorithms in diffusion weighted imaging (DWI) of the head, neck, and pancreas, and T2-TSE of the prostate have also been described [7–9]. The use of AI-based denoising reconstruction has also been described in the adult brain [10, 11]. More recent literature describes the use of AI reconstruction algorithms in the pediatric brain, reporting scan reduction time with synthetic MRI and improved imaging quality on T2-weighted images [12, 13]. Our study contributes to the current literature by adding data regarding the use of an AI reconstruction algorithm on T2W FLAIR in the pediatric brain.

One of the challenges with the application of AI reconstruction algorithms is the presence of “hallucinations,” in which the model generates incorrect data that was not present in the training data [14]. We attempted to address this by specifically examining the reproducibility of pathology and normal variants on the AI-reconstructed images, along with assessing for the presence or absence additional pathologies on the AI-reconstructed images. While we did not document any missed or “hallucinated” pathology on the AI-reconstructed images, more detailed work in this space in future studies, such as detailed evaluation of gyral-sulcal patterns and vascular anatomy, may prove valuable [10]. In the few cases in which image quality on AI-reconstructed images was felt to be poor (*n*=1) or had worse image quality on certain image parameters (*n*=2) in comparison to standard reconstruction, these exams were compromised by motion artifact. When looking at our entire cohort, AI-reconstructed images did not appear to reduce motion artifacts in nearly all patients examined. Similar results were reported in another study examining AI-reconstructed T2-weighted images of the brain [13].

Our study has some limitations. The lack of blinding of the radiologists to the sequence that was being viewed when comparing images can potentially introduce bias, though was difficult to truly avoid given the study radiologists’ knowledge and experience with the clinical protocol. Also, the single scanner, single-institutional nature of this study limits the generalizability of the results to other field strengths, MRI vendors, and institutions. In addition, detailed assessment of the differences in appearance of various pathologies such as gliosis or normal variants including terminal myelination zones was not assessed. We chose not to enroll patients who were being worked up for epilepsy, a protocol which employs 3D FLAIR imaging at our institution. Outside of the enrolled patient with tuberous sclerosis (Fig. 3), none of the other enrolled patients had evidence focal cortical dysplasia; however, the diagnostic utility of AI reconstruction in this particular pathology could be a potential future investigation. Finally, the decision to select only the “strong” denoising algorithm from the 4-point scale provided by the vendor was made because on the pre-study assessment of the sequence feasibility “strong” appeared to have a readily visible difference in T2W FLAIR image quality compared with standard reconstruction, which was more challenging to appreciate at lower denoising levels, and the “maximum” algorithm had an overall appearance too far deviated (appearing “artificial”) from what is expected in conventional clinical T2W FLAIR imaging. We acknowledge that a more detailed examination of the various denoising levels for future studies may be informative.

The use of AI in accelerated compressed sensing shows promise to reduce scan times while preserving imaging quality [10, 15]. MRI of the brain is the most frequently performed MRI exam in our department, and methods allowing for decreased scan time with preserved image quality will be valuable in improving workflow efficiency, patient throughput, scheduling access, and ultimately patient satisfaction in the future. While the ability to use AI reconstruction to decrease scan time in clinical T2W FLAIR imaging by increasing compressed sensing was not explored in the present study, the present findings are encouraging and support the use of AI image reconstruction techniques to improve the quality of clinical pediatric brain MR images with no increase in scan acquisition time.

## Conclusion

AI reconstruction improved T2W FLAIR overall imaging quality and SNR (both quantitative and qualitative) in most patients when compared with standard reconstruction in the pediatric brain. Based on our observations, we believe its use in clinical practice in routine pediatric neuroimaging is safe and feasible.

### Electronic supplementary material

Below is the link to the electronic supplementary material.

Supplementary Material 1

## Data Availability

Derived data supporting the findings of this study are available from the corresponding author on reasonable request.
