# Supplementary material for: Evaluation of T2W FLAIR MR image quality using artificial intelligence image reconstruction techniques in the pediatric brain
Source: Pediatr Radiol. 2024 Jun 18;54(8):1337–43. doi: 10.1007/s00247-024-05968-8 (PMC11254965; doi:10.1007/s00247-024-05968-8)
Supplement: Supplementary file 1 — Supplementary Material 1 [file 247_2024_5968_MOESM1_ESM.docx]

**Supplementary Material 1:**

| Imaging Parameter | Description |
| --- | --- |
| Overall Image Quality | Assessment of which images look better overall taking all imaging facets into account. |
| Subjective SNR | Subjective assessment of the quality of image signal intensity compared to image noise on a given sequence. |
| Diagnostic Preference | Assessment of one’s ability to make the correct diagnosis given the quality of a given sequence. |
| CSF Artifacts | Evaluation of flow artifacts within the CSF (ventricles, cisterns, extra-axial CSF spaces). |
| Motion Artifacts | Assessment of the degree of patient motion degrading the image quality. |
| Susceptibility Artifacts | Degree of signal loss associated with areas of magnetic susceptibility, mainly air in mastoid air cells and paranasal sinuses |
| Grey-White Matter Differentiation | Evaluation of one’s ability to accurately differentiate grey and white matter both in overall signal intensity and along their interfaces. |
| Image Sharpness | Subjective evaluation of spatial resolution of the intracranial structures. |
| Flow Void Visualization | Ability to accurately evaluate the major arterial flow voids (mainly cavernous internal carotid arteries on FLAIR images). |
| Extra-Cranial Structure Visualization | Evaluation of quality of extra-calvarial soft tissues such as the scalp and orbits. |

SNR = signal to noise ratio, CSF = cerebrospinal fluid, FLAIR = fluid attenuated inversion recovery
